# Patient-Reported Outcomes Following Non-Hormonal Treatment of Genitourinary Syndrome of Menopause in Women with Hormone-Dependent Cancer: A Prospective Randomized Pilot Study

**DOI:** 10.3390/healthcare14182959

**Published:** 2026-09-10

**Authors:** Tina Lipovec, Sebastjan Merlo, Ines Cilenšek, Nina Kovacevic

**Affiliations:** 1 Faculty of Medicine, University of Ljubljana, 1000 Ljubljana, Slovenia; tina.lipovec@kcl.ac.uk (T.L.); smerlo@onko-i.si (S.M.); ines.cilensek@mf.uni-lj.si (I.C.); 2 Department of Gynecological Oncology, Institute of Oncology Ljubljana, Zaloška cesta 2, 1000 Ljubljana, Slovenia

**Keywords:** genitourinary syndrome of menopause, cancer survivorship, sexual function, quality of life, Er:YAG laser, hyaluronic acid

## Abstract

**Highlights:**

**What are the main findings?**

- Both active interventions were associated with short-term within-group improvements in VSQ and FSFI scores; without a sham or untreated control, these changes cannot establish intervention-specific efficacy.
- The primary between-group comparison showed no difference in total VSQ change, whereas the exploratory baseline-adjusted total FSFI estimate favored Er:YAG laser therapy; domain-level analyses were exploratory and were not adjusted for multiplicity.

**What are the implications of the main findings?**

- These preliminary comparative findings support further controlled evaluation of non-hormonal strategies for managing genitourinary syndrome of menopause in cancer survivors for whom hormonal therapy may be contraindicated or avoided.
- Routine assessment and treatment of vulvovaginal symptoms and sexual dysfunction should be integrated into comprehensive survivorship care for women with hormone-dependent malignancies.

**Abstract:**

**Background:** Genitourinary syndrome of menopause (GSM) is a common consequence of hypoestrogenism that negatively affects vulvovaginal health, sexual function, and quality of life. Evidence comparing non-hormonal treatment options in women with hormone-dependent malignancies remains limited. This study evaluated patient-reported outcomes following non-ablative Er:YAG laser therapy or hyaluronic acid vaginal gel in women with hormone-dependent cancer and GSM. **Methods:** In this prospective randomized pilot study, 65 women with hormone-dependent malignancies and GSM were randomized to receive either three sessions of non-ablative Er:YAG vaginal laser therapy (*n* = 34) or 0.2% hyaluronic acid vaginal gel applied intravaginally every 72 h for two months (*n* = 31). The primary outcome was change in the total Vulvovaginal Symptom Questionnaire (VSQ) score; change in the total Female Sexual Function Index (FSFI) score was a secondary outcome, and domain-level analyses were exploratory. Outcomes were assessed at baseline and one month after treatment. Wilcoxon signed-rank and Mann–Whitney U tests were used for the principal analyses; adjusted analyses were performed using ANCOVA. The study was retrospectively registered at ClinicalTrials.gov (Identifier: NCT07420647; first posted on 19 February 2026). **Results:** Within-group improvements were observed under both active interventions. The primary between-group comparison showed no difference in total VSQ change (*p* = 0.398). Total FSFI change favored laser therapy in the unadjusted between-group comparison (*p* = 0.034), and the exploratory baseline-adjusted difference was 2.72 points (95% CI, 0.19–5.25; *p* = 0.036). Domain-level analyses were exploratory and were not adjusted for multiplicity. **Conclusions:** In this active-comparator pilot study, total VSQ change was comparable between groups and the adjusted total FSFI estimate favored laser therapy. Without a sham or untreated control, within-group changes cannot establish intervention-specific efficacy; these comparative findings require confirmation in larger controlled trials.

## 1. Introduction

Improvements in cancer diagnosis and treatment have led to a growing population of long-term cancer survivors. Consequently, attention has increasingly shifted from survival alone toward the long-term consequences of cancer and its treatment [1]. Quality of life, sexual health, and functional well-being are now recognized as essential components of comprehensive survivorship care, particularly among women treated for hormone-dependent malignancies [2]. Genitourinary syndrome of menopause (GSM) is one of the most common chronic adverse effects associated with hypoestrogenism. The syndrome encompasses a range of vulvovaginal and lower urinary tract symptoms, including vaginal dryness, irritation, burning, dyspareunia, and sexual dysfunction [3,4]. Unlike vasomotor symptoms, GSM is typically progressive and may substantially impair intimate relationships, self-esteem, sexual functioning, and overall quality of life [4]. Women with hormone-dependent malignancies represent a particularly vulnerable population. Surgical treatment, ovarian suppression, endocrine therapies, and treatment-related hormonal alterations frequently accelerate or exacerbate GSM symptoms. As survival rates continue to improve, the burden of these long-term treatment-related effects is becoming increasingly relevant in routine oncological practice [2,5,6]. Although local oestrogen therapy remains one of the most effective treatments for GSM in the general population, its use in women with hormone-dependent malignancies remains controversial and is frequently avoided because of safety concerns, physician caution, or patient preference [5,7,8]. Consequently, there is a growing need for effective non-hormonal therapeutic strategies capable of improving symptoms while avoiding additional hormonal exposure [9,10]. Among currently available non-hormonal approaches, hyaluronic acid vaginal preparations and vaginal laser therapy have received considerable attention. Hyaluronic acid has demonstrated beneficial hydrating and regenerative effects on vaginal mucosa [11,12], whereas non-ablative Er:YAG laser therapy has been associated with symptom improvement and restoration of vaginal tissue health [13,14,15,16]. However, despite increasing clinical use of both interventions, comparative evidence in women with hormone-dependent malignancies remains limited [17,18]. Although patient-reported outcomes have been evaluated in previous studies, direct comparisons of non-hormonal treatment strategies in women with hormone-dependent cancer remain scarce. Furthermore, relatively limited evidence is available regarding the comparative effects of these interventions on symptom burden and sexual function [17,19]. Understanding comparative changes in patient-reported outcomes is therefore important for evidence-based survivorship care. The aim of this prospective randomized pilot study was to compare treatment-related changes in vulvovaginal symptoms and sexual function between non-ablative Er:YAG laser therapy and hyaluronic acid vaginal gel in women with hormone-dependent malignancies and GSM.

## 2. Materials and Methods

### 2.1. Study Design and Participants

This prospective randomized pilot study was designed to provide preliminary comparative evidence regarding two non-hormonal treatment strategies for genitourinary syndrome of menopause (GSM) in women with hormone-dependent malignancies. The study was conducted at the Institute of Oncology Ljubljana, Slovenia, between April 2025 and April 2026. The first participant was enrolled on 1 April 2025. Eligible participants were women diagnosed with hormone-dependent malignancies who reported symptoms consistent with GSM, including vaginal dryness and associated sexual dysfunction. Participants were required to have serum oestradiol concentrations ≤ 0.11 nmol/L, be aged 65 years or younger, and report sexual activity during the study period. Women with recurrent malignancy, a history of pelvic radiotherapy or brachytherapy, other malignant diseases, or incomplete questionnaire data were excluded from the study. All participants had completed primary cancer treatment before enrolment. Concurrent adjuvant endocrine therapy was not systematically recorded. The study protocol was approved by the National Medical Ethics Committee of the Republic of Slovenia on 26 November 2024 (approval number: 0120-465/2024-2711-3). The trial was retrospectively registered at ClinicalTrials.gov (Identifier: NCT07420647; first posted on 19 February 2026), after participant recruitment had commenced. Ethics approval preceded enrolment, and all participants provided written informed consent before enrolment. Prospective registration was inadvertently overlooked before the first participant was enrolled. The total VSQ score was defined a priori as the primary outcome and the total FSFI score as the secondary outcome before database entry and review of outcome data.

The upper age limit of 65 years was a prespecified pragmatic criterion intended to reduce clinical heterogeneity in this small pilot study and to focus the analysis on sexually active cancer survivors with treatment-associated or relatively early postmenopausal GSM; it was not intended as a biological threshold. Women with previous pelvic radiotherapy or brachytherapy were excluded because radiation can cause vaginal vascular injury, mucosal atrophy, scarring, fibrosis, and stenosis that are pathophysiologically distinct from predominantly hypoestrogenic GSM, potentially introducing substantial clinical heterogeneity and confounding treatment-response assessment.

The study protocol planned the enrolment of at least 60 participants, with at least 30 participants per treatment group, based on an a priori sample-size calculation using a two-sided α of 0.05 and 80% statistical power. The final sample included 65 participants.

By the date on which the ClinicalTrials.gov record was first posted, 55 of the 65 participants had already been enrolled and randomized. No separate, standalone statistical analysis plan was prepared. The broad statistical approach had been documented before participant enrolment in the ethics-approved research plan, and the total VSQ and total FSFI outcome hierarchy had been defined before database entry and examination of outcome data.

### 2.2. Randomization and Interventions

Participants were assigned in a 1:1 ratio to either non-ablative Er:YAG laser therapy or hyaluronic acid vaginal gel. An independent member of the research team who was not involved in participant enrolment, treatment delivery, or outcome assessment generated and retained the computer-based block-randomization sequence, which used randomly varying block sizes. After eligibility had been confirmed, informed consent obtained, and the participant enrolled by the study investigators, the independent team member disclosed the next allocation. The investigators enrolling participants therefore had no access to the allocation sequence and could not foresee the upcoming assignment. Because the interventions were visibly different, participants and the treating gynecologist were not blinded. The statistical analyst was blinded to treatment identity: raw data were provided without participant names, and the treatment groups were coded as Group 1 and Group 2 without disclosure of which code represented laser or hyaluronic acid treatment. The study directly compared two active non-hormonal treatments; no placebo, sham-laser, or untreated control group was included. Participants assigned to the laser group received three outpatient intravaginal treatments at four-week intervals using a 2940 nm Er:YAG laser operated in non-ablative SMOOTH mode. Each session followed the manufacturer-defined two-step RenovaLase protocol using a vaginal handpiece. The RenovaLase 1 preset was used to treat the entire vaginal canal with three passes at a fluence of 1.7 J/cm^2^ and a repetition frequency of 3.3 Hz, and the RenovaLase 2 preset was used to treat the vaginal vestibule and introitus with three passes at a fluence of 10.0 J/cm^2^ and a repetition frequency of 1.6 Hz. The preset parameters were used without manual modification. The same protocol was applied at all three sessions. Participants assigned to the comparator group received a vaginal gel containing 0.2% hyaluronic acid. Participants self-administered the gel directly into the vaginal canal using a vaginal applicator (intravaginal application, not injection) every 72 h for two consecutive months according to the manufacturer’s instructions. At the initial study visit, the application procedure was explained and demonstrated to each participant. Participants were instructed to apply the gel every 72 h and, if a scheduled application was missed, to apply it as soon as they remembered. Participants were also instructed to maintain a treatment diary.

The laser procedures were performed using the TimeWalker IntimaLaser Pro platform (Fotona d.o.o., Ljubljana, Slovenia), and the manufacturer-defined RenovaLase treatment protocol. All laser procedures were performed by the same gynecologist trained in the use of this platform and treatment protocol. The hyaluronic acid product was HYALOFEMME^®^ vaginal gel containing 0.2% Hydeal-D (Fidia Farmaceutici S.p.A., Abano Terme, Italy). Use of additional vaginal moisturizers, lubricants, local hormonal therapies, pelvic-floor therapy, or other GSM-directed interventions was prohibited during the study period.

### 2.3. Outcome Assessment

Outcome assessments were performed at baseline and one month after completion of treatment. No 3- or 6-month outcome assessments were collected for this pilot analysis. The primary outcome was the change from baseline in the total VSQ score. Change in the total FSFI score was a secondary outcome, and analyses of individual VSQ and FSFI domains were exploratory. Vulvovaginal symptoms were assessed using a 21-item Vulvovaginal Symptoms Questionnaire (VSQ), originally developed and validated by Erekson et al. for postmenopausal women. The VSQ comprises four domains—symptoms, emotional impact, life impact, and sexual impact—and higher scores indicate greater symptom burden; item 17 is a branching item and is not included in the score [20]. Sexual function was assessed using the 19-item Female Sexual Function Index (FSFI), developed and validated by Rosen et al. The FSFI comprises six domains: desire, arousal, lubrication, orgasm, satisfaction, and pain; higher scores indicate better sexual function [21].

Both questionnaires were completed independently by participants, using Slovenian-language versions. The Slovenian VSQ was prepared by forward-translation and back-translation. The Slovenian FSFI had undergone linguistic validation using translation from English into Slovenian and back-translation [22].

### 2.4. Statistical Analysis

Statistical analyses were performed using IBM SPSS Statistics version 26.0 (IBM Corp., Armonk, NY, USA). The distribution of continuous variables was assessed using the Shapiro–Wilk test. As questionnaire scores demonstrated predominantly non-normal distributions, non-parametric statistical methods were used for the unadjusted analyses. The between-group comparison of change in total VSQ was the single primary analysis. Change in the total FSFI score was evaluated as a secondary outcome; domain-level comparisons and all adjusted models were exploratory. Within-group changes were summarized descriptively and tested using the Wilcoxon signed-rank test, but comparative inference was based on between-group differences in change or baseline-adjusted treatment effects. Between-group differences in treatment-related change were assessed using the Mann–Whitney U test. Effect sizes were calculated as r = Z/√N and interpreted according to conventional thresholds (0.10 = small, 0.30 = moderate, and 0.50 = large). No adjustment for multiplicity was applied to the secondary total FSFI comparison or to the exploratory within-group, domain-level, adjusted ANCOVA, correlation, and regression analyses. All *p* values from these secondary and exploratory analyses are nominal and should be interpreted as hypothesis-generating rather than confirmatory. ANCOVA was used as an exploratory analysis to compare follow-up scores between the treatment groups while adjusting for the corresponding baseline score. In each model, the follow-up score was entered as the dependent variable, treatment group as the fixed factor, and the corresponding baseline score as the covariate. Baseline adjustment was selected to account for individual differences at study entry and to improve the precision of the between-group comparison. Although several unadjusted outcome scores were not normally distributed, the distributional assumption of ANCOVA primarily concerns model residuals rather than the raw outcome scores. The similarly sized treatment groups also supported the robustness of the analysis.

Model assumptions were evaluated separately for each ANCOVA model. Linearity was examined by adding a quadratic baseline term, homogeneity of regression slopes by testing the group-by-baseline interaction, homoscedasticity using the Breusch–Pagan test, residual distribution using the Jarque–Bera test, and potentially influential observations using Cook’s distance. The ANCOVA analyses were exploratory and did not replace the primary nonparametric comparisons.

In these models, follow-up scores were entered as dependent variables, treatment group was included as the fixed factor, and baseline scores were entered as covariates. Assumptions of ANCOVA, including homogeneity of regression slopes, were assessed before model estimation. Associations between changes in vulvovaginal symptoms and sexual function were examined using Spearman rank correlation coefficients. Exploratory multivariable linear regression analyses were performed to evaluate whether treatment modality, age, cancer type, and serum oestradiol levels independently predicted treatment-related changes. Changes in VSQ and FSFI scores were entered as dependent variables in separate regression models. All statistical tests were two-sided, and statistical significance was defined as *p* < 0.05.

## 3. Results

### 3.1. Study Population

**Figure 1:**
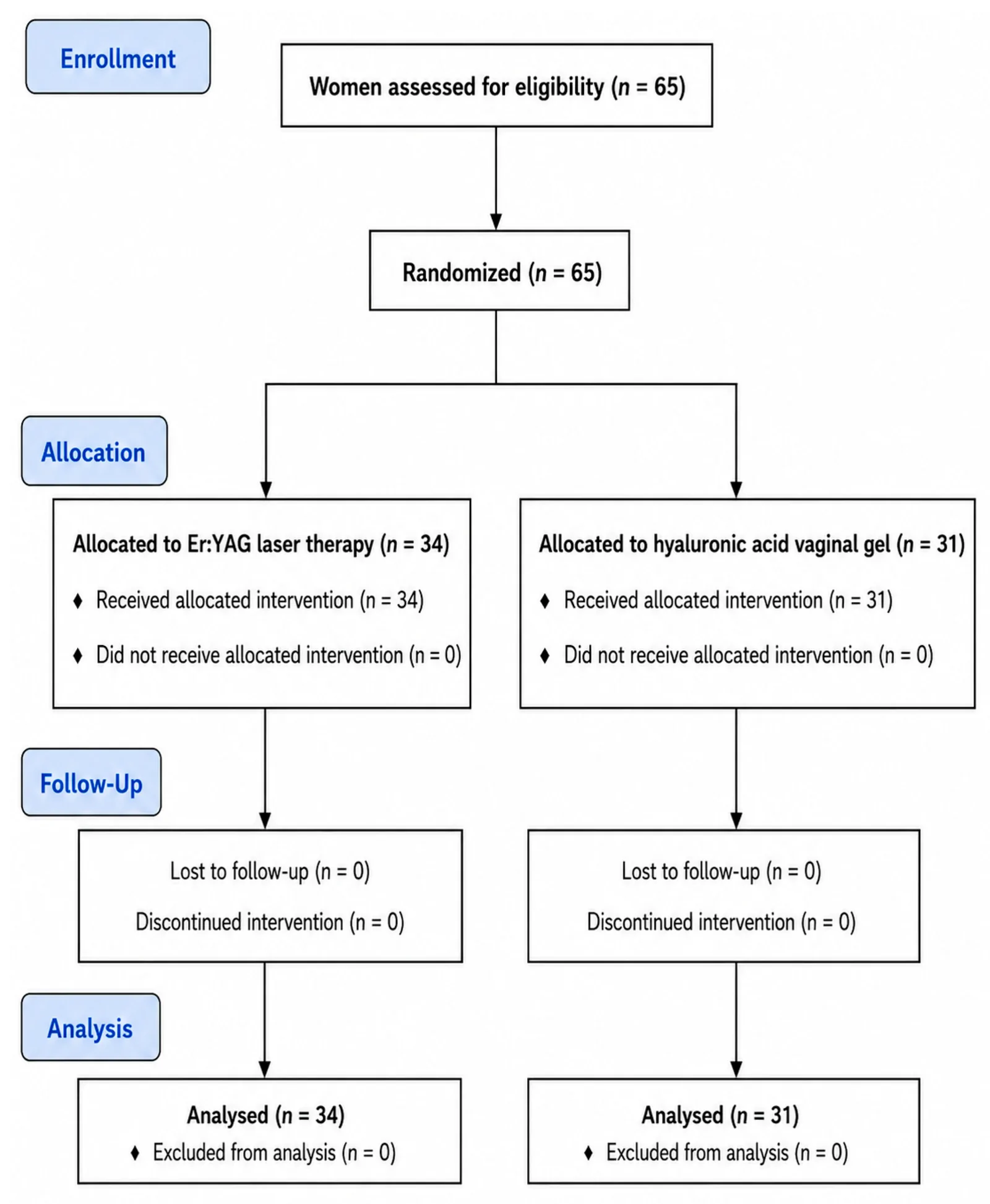
Flow diagram of participant enrolment, randomization, follow-up, and analysis. The diagram begins with women formally assessed and entered in the study log; informal pre-screening before formal assessment was not recorded.

A total of 65 women with hormone-dependent malignancies and symptoms of genitourinary syndrome of menopause were enrolled in this prospective randomized pilot study. Thirty-four participants were allocated to the Er:YAG laser group and thirty-one to the hyaluronic acid group. All participants completed baseline and follow-up assessments and were included in the final analysis. Baseline demographic and clinical characteristics of the study population are presented in Table 1. No statistically significant differences between treatment groups were observed at baseline. Participant enrolment, randomization, follow-up, and analysis are summarized in the CONSORT flow diagram (Figure 1).

The study log began at formal eligibility assessment. Sixty-five women were formally assessed that all met the eligibility criteria, provided consent, and were randomized. The numbers of women who may have undergone informal pre-screening, declined participation before formal assessment, or were excluded before entry into the study log were not recorded and could not be reconstructed retrospectively.

All 34 participants allocated to laser treatment completed all three scheduled sessions, and no laser sessions were missed. All 31 participants allocated to hyaluronic acid treatment reported completing the full prescribed treatment course and all planned gel applications. Participant-level self-reported treatment completion was therefore 100% in both groups. However, because diary-derived dose-level data were not available for quantitative analysis, the exact timing of any delayed gel applications and the proportion administered precisely at the prescribed 72 h intervals could not be independently verified. Adverse events were not prospectively or systematically collected. No serious treatment-related event or treatment discontinuation came to the investigators’ attention; however, because participants were not systematically questioned and adverse events were not formally recorded, the occurrence and frequency of minor events such as pain, bleeding, discharge, infection, or irritation could not be reliably determined.

### 3.2. Vulvovaginal Symptoms

Changes in vulvovaginal symptoms assessed using the VSQ are presented in Table 2.

Within the laser group, the total VSQ score decreased from baseline (Wilcoxon Z = −5.043, *p* < 0.001, r = 0.87), and a corresponding decrease was observed within the hyaluronic acid group (Wilcoxon Z = −4.790, *p* < 0.001, r = 0.86). These within-group findings describe change under each active intervention but do not establish intervention-specific efficacy in the absence of a sham or untreated control. The primary between-group comparison showed no statistically significant difference in total VSQ change (Mann–Whitney U = 463.0, Z = −0.845, *p* = 0.398; r = 0.11). The Hodges–Lehmann estimate of the between-group difference in change (laser minus hyaluronic acid) was −1.0 points (95% CI, −2.0 to 1.0).

### 3.3. Sexual Function

Changes in sexual function assessed using the Female Sexual Function Index (FSFI) are presented in Table 3.

Within-group improvements in total FSFI and its domains were observed under both active interventions (all Wilcoxon *p* < 0.001). The secondary between-group comparison of total FSFI change favored laser (Mann–Whitney U = 365.5, Z = −2.121, *p* = 0.034; r = 0.26). The *p* value for this secondary outcome is nominal because no multiplicity adjustment was applied. Among the 10 exploratory VSQ and FSFI domain-level comparisons, arousal had a nominal *p* value of 0.006. This domain-level result remains exploratory. Data are presented in Table 4.

### 3.4. Adjusted Analyses

To account for potential baseline differences, analyses of covariance (ANCOVA) were performed using baseline scores as covariates (Table 5 and Table 6).

After adjustment for baseline scores, the adjusted total VSQ mean difference (laser minus hyaluronic acid) was −0.52 points (95% CI, −1.96 to 0.91; F = 0.536, *p* = 0.467, partial η^2^ = 0.009). The adjusted total FSFI mean difference favored laser by 2.72 points (95% CI, 0.19 to 5.25; F = 4.610, *p* = 0.036, partial η^2^ = 0.069). Adjusted mean follow-up FSFI scores were 25.24 for laser and 22.52 for hyaluronic acid. No multiplicity adjustment was applied to the adjusted ANCOVA analyses; all associated *p* values are nominal, and these findings should be interpreted as exploratory.

Model diagnostics did not indicate material departures from the evaluated assumptions in the total-score ANCOVA models. For the total VSQ model, there was no evidence of non-homogeneous regression slopes (group-by-baseline interaction, *p* = 0.579), nonlinearity (quadratic baseline term, *p* = 0.503), heteroscedasticity (Breusch–Pagan test, *p* = 0.121), or non-normal residuals (Jarque–Bera test, *p* = 0.414). The corresponding diagnostic tests for the total FSFI model were also non-significant (all *p* ≥ 0.062). At the domain level, the Breusch–Pagan test indicated possible heteroscedasticity for VSQ Symptoms (*p* = 0.015), VSQ Emotional Impact (*p* = 0.037), VSQ Life Impact (*p* = 0.021), and FSFI Pain (*p* = 0.033). The VSQ Emotional Impact model also showed evidence of non-homogeneous regression slopes (*p* = 0.037), nonlinearity (*p* = 0.006), and non-normal residuals (*p* < 0.001). Evidence of nonlinearity was additionally observed for FSFI Lubrication (*p* = 0.047), Orgasm (*p* = 0.023), and Satisfaction (*p* = 0.041), while non-normal residuals were observed for FSFI Arousal (*p* < 0.001). The maximum Cook’s distance across the evaluated models was 0.270. These domain-level departures reinforce that the adjusted domain-level estimates should be regarded as exploratory and interpreted cautiously.

### 3.5. Exploratory Analyses

The reduction in total VSQ score was moderately correlated with improvement in total FSFI score (Spearman ρ = 0.373, *p* = 0.002). This exploratory association does not establish a causal pathway. The multivariable model for VSQ change was not statistically significant (R^2^ = 0.020, *p* = 0.873), and none of the evaluated variables independently predicted symptom improvement. In the exploratory FSFI model, treatment modality was associated with improvement (β = 0.266, *p* = 0.038), whereas age, cancer type, and oestradiol levels were not significantly associated with FSFI change. These exploratory regression findings should be interpreted as hypothesis-generating. No multiplicity adjustment was applied to the correlation or regression analyses; all associated *p* values are nominal and should be interpreted as hypothesis-generating.

## 4. Discussion

This randomized active-comparator pilot study evaluated changes in patient-reported vulvovaginal symptoms and sexual function under two non-hormonal interventions. The primary comparison showed no between-group difference in total VSQ change. The exploratory baseline-adjusted secondary comparison favored laser for total FSFI, with a modest adjusted mean difference. Within-group improvements under both interventions are descriptive and cannot demonstrate efficacy without a sham or untreated control. Domain-level and multivariable findings remain exploratory.

Because baseline total VSQ scores showed a possible imbalance between groups (median 9.0 in the laser group and 11.0 in the hyaluronic acid group; *p* = 0.052), the baseline-adjusted estimates were considered particularly important when interpreting comparative treatment effects.

These findings are clinically relevant because GSM represents one of the most common and persistent survivorship challenges among women treated for hormone-dependent malignancies [2,5]. As advances in cancer treatment continue to improve long-term survival, the management of treatment-related symptoms has become increasingly important. Vulvovaginal symptoms, dyspareunia, and sexual dysfunction may substantially affect quality of life, intimate relationships, emotional well-being, and overall survivorship experience [6,20,23]. Despite their clinical importance, these issues often remain underrecognized and undertreated within routine oncological care [24].

Both groups showed marked short-term within-group changes, consistent with prior reports of symptom improvement during non-hormonal management [9,11,12,17,19]. Gold et al. [17] likewise reported improvements under vaginal laser and hyaluronic acid in breast cancer survivors. However, within-group change in an active-comparator study may reflect intervention effects, expectations, clinical attention, regression to the mean, or natural fluctuation. The present findings therefore support continued comparative evaluation rather than a conclusion that either intervention is effective relative to no treatment.

The adjusted total FSFI estimate favored laser by 2.72 points (95% CI, 0.19 to 5.25), whereas the adjusted total VSQ mean difference was −0.52 points (95% CI, −1.96 to 0.91). Domain-level *p* values were not adjusted for multiplicity and should not be used to localize a comparative effect to a specific domain. From a clinical perspective, 16 of 34 women in the laser group (47.1%) and 10 of 31 women in the hyaluronic acid group (32.3%) had an FSFI total score above the established diagnostic cutoff of 26.55 at follow-up [25]. However, this threshold is a diagnostic cutoff for female sexual dysfunction rather than a validated minimal clinically important difference for treatment response. These proportions therefore provide descriptive clinical context but should not be interpreted as definitive treatment-responder rates. Previous histologic and clinical literature has proposed that non-ablative Er:YAG laser may be associated with collagen remodeling, vascular changes, epithelial elasticity, and mucosal restoration [13,14,15,16,26]. These are literature-derived mechanistic hypotheses only: the present study measured patient-reported outcomes and did not assess tissue structure, vascularization, histology, or mediation. The adjusted 2.72-point difference and partial η^2^ of 0.069 indicate a modest statistical effect, but no validated minimal clinically important difference for the between-treatment FSFI difference in this population was available; its clinical importance therefore remains uncertain. No biological mechanism was directly demonstrated in this study.

The exploratory correlation between improvement in VSQ and FSFI scores is compatible with a relationship between symptom burden and sexual function, but it cannot establish that symptom relief caused the change in sexual function. Its moderate magnitude also reflects that sexual health in cancer survivors is multidimensional and may be influenced by relational, psychological, treatment-related, and other factors beyond vulvovaginal symptoms [2,6,21].

From a clinical perspective, these preliminary comparative estimates may inform shared decision-making when hormonal treatment is contraindicated or avoided, but they do not establish either active intervention as effective compared with no treatment. Choice between approaches should consider patient preference, availability, treatment burden, expected adherence, safety, and uncertainty regarding long-term benefit [2,7,8,9,27].

The therapeutic spectrum for GSM also includes regular vaginal moisturizers and lubricants, pelvic-floor interventions, and, for carefully selected patients, local hormonal approaches considered through individualized discussion with the oncology team. Multimodal strategies may combine approaches; for example, combined intravaginal estriol and Kegel exercises have been evaluated in postmenopausal vulvovaginal atrophy [28]. However, evidence from general postmenopausal populations cannot be directly extrapolated to survivors of hormone-dependent cancer, for whom treatment choice requires individualized assessment of symptoms, preferences, contraindications, and oncological context.

Safety and tolerability require separate consideration. Intravaginal hyaluronic acid is generally well tolerated, although local irritation, burning, itching, or increased vaginal discharge may occur [11,12,17,18]. Non-ablative Er:YAG treatment may be followed by transient warmth or discomfort, watery discharge, spotting, dysuria, or local irritation; available evidence remains insufficient to establish uncommon or long-term harms, particularly in cancer survivors [13,14,15,16,17,18]. The present pilot study was designed primarily to assess patient-reported outcomes and was not powered to compare uncommon adverse events. These distinctions should be discussed during shared decision-making, and patients receiving laser treatment should be monitored according to the device protocol and local clinical governance. In the present study, adverse events were not prospectively or systematically collected. Although no serious treatment-related event or treatment discontinuation came to the investigators’ attention, minor adverse events cannot be excluded or quantified reliably.

The strengths of the present study include its prospective randomized design, the use of established patient-reported outcome measures, complete follow-up of all randomized participants, and the direct comparison of two active non-hormonal treatment strategies within a relatively homogeneous population of women with hormone-dependent malignancies. In addition, our findings contribute to the growing body of evidence supporting non-hormonal management of GSM by demonstrating short-term improvements in both vulvovaginal symptoms and sexual function. Several limitations should be acknowledged. This was a pilot study with a relatively small sample size, limiting statistical power and the ability to detect smaller between-group differences. The study was conducted at a single institution, potentially limiting generalizability. Furthermore, the study population consisted predominantly of women with breast cancer, while only a small proportion had endometrial cancer. Therefore, the findings should be interpreted cautiously when extrapolating the results to women with other hormone-dependent malignancies. Follow-up was limited to one month after treatment completion, and no 3- or 6-month assessments were available; the durability of benefit therefore cannot be determined. The absence of a placebo, sham-laser, or untreated control group prevents separation of treatment-specific effects from placebo, attention, and other non-specific effects. Trial registration occurred after recruitment had begun. Although ethics approval preceded enrolment and the registration timing is reported transparently, prospective registration would have strengthened protection against selective reporting. The study was not designed or powered to compare uncommon or long-term adverse events between interventions. Blinding of participants and treating clinicians was not feasible because of the nature of the interventions, which may have influenced patient-reported outcomes. No multiplicity adjustment was applied to the secondary total FSFI comparison or to the exploratory within-group, domain-level, adjusted ANCOVA, correlation, and regression analyses. Their *p* values are nominal and should be interpreted cautiously as hypothesis-generating rather than confirmatory. Diagnostic evaluation supported interpretation of the total-score ANCOVA models, whereas several exploratory domain-level models showed evidence of heteroscedasticity, nonlinearity, non-normal residuals, or, in one model, non-homogeneous regression slopes. These departures reinforce the need to interpret the adjusted domain-level estimates cautiously and as hypothesis-generating. All participants had completed primary cancer treatment before enrolment, concurrent adjuvant endocrine therapy was not systematically recorded and its potential influence on symptoms and treatment response could therefore not be evaluated. The exploratory regression analyses should also be interpreted cautiously because of the limited sample size relative to the number of predictors examined. Because 61 of the 65 participants had breast cancer and only four had endometrial cancer, the findings primarily reflect breast cancer survivors and should not be generalized broadly to all hormone-dependent malignancies. Requiring sexual activity during the study may also have introduced selection bias and limits applicability to survivors who are not currently sexually active. The pragmatic upper age limit of 65 years further limits generalizability to older survivors. Detailed oncological and endocrine-treatment variables, including current aromatase-inhibitor or tamoxifen use, ovarian suppression, mechanism and timing of menopause, and time since cancer treatment, were not available in the analytic dataset. Adherence to the self-administered gel regimen could not be quantified, and adverse events were not systematically collected. Informal pre-screening before formal eligibility assessment was not recorded, so the numbers who declined or were excluded at that stage could not be reconstructed.

Many survivorship interventions focus primarily on disease surveillance and recurrence detection. The present comparative estimates reinforce the importance of studying symptom-oriented care and sexual health as components of survivorship, while avoiding causal claims that the active-comparator design cannot support [29]. Future multicentre trials should include an appropriate placebo or sham comparator, prospectively register outcomes, systematically capture adverse events, and assess outcomes at 3 and 6 months or longer to determine the durability and comparative safety of treatment effects. Future studies should also systematically record concurrent endocrine therapy, prespecify multiplicity control and clinically meaningful responder thresholds, and include direct tissue or vascular measurements when testing mechanistic hypotheses.

## 5. Conclusions

In this randomized active-comparator pilot study, the primary total VSQ change did not differ between groups, whereas the exploratory baseline-adjusted total FSFI estimate favored laser therapy. Domain-level findings were exploratory and were not adjusted for multiplicity. Because the study lacked a placebo or sham comparator and included only short-term follow-up, these findings should be interpreted as preliminary and require confirmation in larger, longer-term controlled trials. The results support further rigorous evaluation of non-hormonal GSM management and routine attention to sexual health and quality of life in women living after hormone-dependent cancer. Adequately powered multicentre randomized trials with longer-term follow-up and, ideally, sham-controlled designs are required to confirm these preliminary findings.

## Figures and Tables

**Table 1:** Baseline Characteristics of the Study Population According to Treatment Group.

Variable	Laser (*n* = 34)	Hyaluronic Acid (*n* = 31)	*p*-Value
Age (years)	52.0 (45.75–56.25)	48.0 (42.0–54.0)	0.146
Education level, No (%)			0.508
• Primary school	0 (0.0)	2 (6.5)	
• Secondary school	12 (35.3)	9 (29.0)	
• College degree	7 (20.6)	8 (25.8)	
• University degree	12 (35.3)	8 (25.8)	
• PhD degree	3 (8.8)	4 (12.9)	
Marital status, No (%)			0.843
• Married	23 (67.6)	19 (61.3)	
• Divorced	2 (5.9)	2 (6.5)	
• Cohabiting	7 (20.6)	9 (29.0)	
• Single	2 (5.9)	1 (3.2)	
Employment status, No (%)			0.645
• Employed	26 (76.5)	26 (83.9)	
• Self-employed	3 (8.8)	3 (9.7)	
• Unemployed	4 (11.8)	1 (3.3)	
• Student	1 (2.9)	1 (3.2)	
Place of residence, No (%)			0.518
• Urban area (>3000 inhabitants)	22 (64.7)	21 (67.7)	
• Rural area (<3000 inhabitants)	12 (35.3)	10 (32.3)	
Site of malignancy			0.348
Breast cancer, No (%)	31 (91.2)	30 (96.8)	
Endometrial cancer, No (%)	3 (8.8)	1 (3.2)	
Serum oestradiol (nmol/L), median (Q1–Q3)	0.02 (0.02–0.03)	0.02 (0.02–0.02)	0.086
Baseline VSQ Total, median (Q1–Q3)	9.0 (7.0–11.3)	11.0 (8.0–13.0)	0.052
Baseline FSFI Total, median (Q1–Q3)	14.4 (9.5–17.8)	15.1 (10.6–19.1)	0.906

Abbreviations: VSQ, Vulvovaginal Symptom Questionnaire; FSFI, Female Sexual Function Index. Continuous variables were compared using the Mann–Whitney U test. Categorical variables were compared using Pearson’s chi-square test.

**Table 2:** Changes in Vulvovaginal Symptoms (VSQ) Following Treatment According to Treatment Group.

Group	Outcome	Baseline, Median (Q1–Q3)	Follow-Up, Median (Q1–Q3)	Z	*p*	r
Laser (*n* = 34)	VSQ Total	9.0 (7.0–11.3)	3.5 (1.0–7.0)	−5.043	<0.001	0.87
	Symptoms	3.0 (1.75–4.0)	1.0 (0.75–2.0)	−4.452	<0.001	0.76
	Emotional Impact	1.0 (0.0–2.25)	0.0 (0.0–1.0)	−3.522	<0.001	0.60
	Life Impact	2.0 (1.0–3.0)	0.0 (0.0–2.0)	−3.384	<0.001	0.58
	Sexual Impact	3.0 (3.0–4.0)	2.0 (0.0–3.0)	−4.372	<0.001	0.75
Hyaluronic acid (*n* = 31)	VSQ Total	11.0 (8.0–13.0)	5.0 (2.0–8.0)	−4.790	<0.001	0.86
	Symptoms	4.0 (2.0–5.0)	1.0 (1.0–2.0)	−4.348	<0.001	0.78
	Emotional Impact	1.0 (1.0–3.0)	0.0 (0.0–2.0)	−3.764	<0.001	0.68
	Life Impact	2.0 (1.0–3.0)	1.0 (0.0–3.0)	−2.983	0.003	0.54
	Sexual Impact	3.0 (3.0–4.0)	2.0 (0.0–3.0)	−4.139	<0.001	0.74

Abbreviations: VSQ, Vulvovaginal Symptom Questionnaire; r, effect size.

**Table 3:** Changes in Sexual Function (FSFI) Following Treatment According to Treatment Group.

Group	Outcome	Baseline, Median (Q1–Q3)	Follow-Up, Median (Q1–Q3)	Z	*p*	r
Laser (*n* = 34)	Desire	1.8 (1.2–2.4)	3.3 (2.4–3.6)	−4.417	<0.001	0.76
	Arousal	2.25 (1.5–3.3)	4.3 (3.6–5.1)	−4.476	<0.001	0.77
	Lubrication	1.8 (1.2–3.4)	4.3 (3.1–5.5)	−4.542	<0.001	0.78
	Orgasm	2.6 (1.2–3.8)	5.2 (4.0–5.6)	−4.676	<0.001	0.80
	Satisfaction	3.0 (1.9–4.1)	4.8 (4.0–5.6)	−4.642	<0.001	0.80
	Pain	1.4 (1.2–2.5)	4.4 (3.6–5.6)	−4.634	<0.001	0.79
	FSFI Total	14.4 (9.5–17.8)	26.1 (21.9–29.9)	−4.984	<0.001	0.85
Hyaluronic acid (*n* = 31)	Desire	1.8 (1.2–3.0)	3.0 (2.4–3.6)	−3.741	<0.001	0.67
	Arousal	2.7 (1.5–3.6)	3.6 (3.0–4.5)	−3.978	<0.001	0.71
	Lubrication	2.1 (1.2–3.3)	3.6 (3.0–4.8)	−4.710	<0.001	0.85
	Orgasm	2.0 (1.2–3.6)	3.2 (2.4–5.2)	−4.338	<0.001	0.78
	Satisfaction	2.8 (2.0–4.0)	4.8 (2.8–5.6)	−4.338	<0.001	0.78
	Pain	2.0 (1.2–2.8)	4.4 (3.6–4.8)	−4.636	<0.001	0.83
	FSFI Total	15.1 (10.6–19.1)	23.2 (16.9–27.7)	−4.842	<0.001	0.87

Abbreviations: FSFI, Female Sexual Function Index; r, effect size.

**Table 4:** Comparison of Treatment-Related Changes in VSQ and FSFI Outcomes Between Groups.

Variable	Hyaluronic Acid (*n* = 31) Mean Rank	Laser (*n* = 34) Mean Rank	Mann–Whitney U	Z	*p*
Δ VSQ Total	35.06	31.12	463	−0.845	0.398
Δ FSFI Total	27.79	37.75	365.5	−2.121	0.034
Δ Symptoms	30.65	35.15	454	−0.977	0.329
Δ Emotional Impact	31.61	34.26	484	−0.588	0.556
Δ Life Impact	33.53	32.51	510.5	−0.223	0.823
Δ Sexual Impact	30.76	35.04	457.5	−0.934	0.350
Δ Desire	30.77	35.03	458	−0.932	0.351
Δ Arousal	26.31	39.10	319.5	−2.741	0.006
Δ Lubrication	29.23	36.44	410	−1.542	0.123
Δ Orgasm	29.34	36.34	413.5	−1.498	0.134
Δ Satisfaction	29.11	36.54	406.5	−1.592	0.111
Δ Pain	30.81	35.00	459	−0.898	0.369

Abbreviations: Δ, change from baseline to follow-up; FSFI, Female Sexual Function Index; VSQ, Vulvovaginal Symptom Questionnaire. Because higher VSQ scores indicate greater symptom burden, whereas higher FSFI scores indicate better sexual function, Δ was defined so that positive values consistently indicate improvement: Δ VSQ = baseline − follow-up for all VSQ outcomes, and Δ FSFI = follow-up − baseline for all FSFI outcomes.

**Table 5:** Adjusted Analyses of Treatment Outcomes Using Analysis of Covariance (ANCOVA) for FSFI.

Outcome	Adjusted Mean (HA) (95% CI)	Adjusted Mean (Laser) (95% CI)	Mean Difference (Laser–HA) (95% CI)	F	*p*-Value	Partial η^2^
Desire	2.92 (2.62–3.22)	3.04 (2.75–3.32)	0.11 (−0.30–0.53)	0.306	0.582	0.005
Arousal	3.51 (3.15–3.88)	4.19 (3.84–4.54)	0.68 (0.18–1.18)	7.283	0.009	0.105
Lubrication	3.80 (3.36–4.23)	4.26 (3.84–4.67)	0.46 (−0.14–1.06)	2.350	0.130	0.037
Orgasm	3.87 (3.45–4.28)	4.62 (4.22–5.02)	0.75 (0.17–1.33)	6.735	0.012	0.098
Satisfaction	4.30 (3.97–4.64)	4.76 (4.44–5.08)	0.45 (−0.005–0.914)	3.915	0.052	0.059
Pain	4.18 (3.72–4.63)	4.34 (3.90–4.77)	0.16 (−0.46–0.79)	0.263	0.610	0.004
FSFI Total	22.52 (20.69–24.35)	25.24 (23.49–26.99)	2.72 (0.19–5.25)	4.610	0.036	0.069

Abbreviations: FSFI, Female Sexual Function Index; HA, Hyaluronic Acid; CI, Confidence interval; η^2^, eta squared. Values are estimated marginal means with 95% confidence intervals. Mean differences are calculated as laser minus hyaluronic acid. Domain-level analyses were exploratory and were not adjusted for multiplicity.

**Table 6:** Adjusted Analyses of Treatment Outcomes Using Analysis of Covariance (ANCOVA) for VSQ.

Outcome	Adjusted Mean (HA) (95% CI)	Adjusted Mean (Laser) (95% CI)	Mean Difference (Laser–HA) (95% CI)	F	*p*-Value	Partial η^2^
Symptoms	1.42 (1.03–1.82)	1.23 (0.86–1.60)	−0.19 (−0.74–0.35)	0.515	0.476	0.008
Emotional Impact	0.78 (0.44–1.12)	0.52 (0.20–0.85)	−0.26 (−0.74–0.22)	1.183	0.281	0.019
Life Impact	1.26 (0.84–1.69)	0.96 (0.56–1.37)	−0.30 (−0.90–0.29)	1.024	0.315	0.016
Sexual Impact	1.53 (1.04–2.02)	1.69 (1.22–2.16)	0.16 (−0.53–0.85)	0.209	0.649	0.003
VSQ Total	4.96 (3.95–5.98)	4.44 (3.47–5.41)	−0.52 (−1.96–0.91)	0.536	0.467	0.009

Abbreviations: VSQ, Vulvovaginal Symptom Questionnaire; HA, Hyaluronic Acid; CI, Confidence interval; η^2^, eta squared. Values are estimated marginal means with 95% confidence intervals. Mean differences are calculated as laser minus hyaluronic acid. Domain-level analyses were exploratory and were not adjusted for multiplicity.

## Data Availability

The de-identified dataset is publicly available in Zenodo (version 1) at https://doi.org/10.5281/zenodo.21823578.

## Abbreviations

The following abbreviations are used in this manuscript:

ANCOVA	Analysis of Covariance
FSFI	Female Sexual Function Index
GSM	Genitourinary Syndrome of Menopause
HA	Hyaluronic Acid
VSQ	Vulvovaginal Symptom Questionnaire
